# Chemokine Networks in Cutaneous T Cell Lymphoma: Tumor Microenvironment Remodeling and Therapeutic Targets

**DOI:** 10.3390/cimb48010079

**Published:** 2026-01-13

**Authors:** Zihao Yu, Fei Li, Ying Quan, Weijian Hu, Ping Zhang, Xin Xie

**Affiliations:** 1 College of Life Sciences, Northwest University, Xi’an 710127, China; 2 Department of Dermatology, Air Force Medical University, Air Force Medical Center, PLA, Beijing 100142, China

**Keywords:** cutaneous T-cell lymphoma (CTCL), chemokines, tumor microenvironment (TME), immune evasion, targeted therapy

## Abstract

Cutaneous T-cell lymphoma (CTCL) is a heterogeneous malignancy characterized by the proliferation of skin-homing CD4^+^ T cells and profound immune dysregulation within the tumor microenvironment (TME). This review synthesizes evidence on chemokine–receptor networks that govern malignant T-cell trafficking among blood, skin, and lymph nodes, the formation of immunosuppressive niches, and clinically actionable biomarker candidates. Among the best-supported axes, CCL17/CCL22–CCR4 and CCL27/CCL28–CCR10 mediate skin tropism, CCL19/CCL21–CCR7 contributes to lymph node homing, and CXCL12–CXCR4 supports skin trafficking and is associated with disease progression. In contrast, CCR2/CCR5/CCR6/CCR8-centered circuits and CXCR3/CXCR5 pathways are emerging regulators of myeloid recruitment, regulatory T-cell accumulation, and context-dependent immune activation. Therapeutically, agents targeting chemokine pathways, most notably the CCR4 monoclonal antibody Mogamulizumab, have demonstrated clinical efficacy, while emerging inhibitors of CCR6, CCR5, and CXCR4 offer promising avenues for intervention. We further highlight how recent single-cell and other high-dimensional omics studies refine cell-type–specific chemokine sources and receptor expression, enabling more precise mapping of chemokine-driven intercellular communication programs in CTCL TME remodeling and better prioritization of therapeutic targets and biomarkers.

## 1. Introduction

Cutaneous T-cell lymphoma (CTCL), a rare type of skin tumor, is a primary non-Hodgkin lymphoma that is mainly caused by the malignant clonal proliferation of skin-resident CD4^+^ T cells [1]. CTCL accounts for approximately 65–75% of all primary cutaneous lymphomas [2,3]. According to data from the World Health Organization’s European Organization for Research and Treatment of Cancer (WHO-EORTC), with the improvement of clinical diagnostic levels, the incidence of CTCL has increased from approximately six to nine cases per million people in 2002 [3]. CTCL shows high heterogeneity in its clinical manifestations and biological characteristics, with the disease spectrum ranging from slowly progressing localized erythema to highly aggressive forms with systemic involvement [3]. Furthermore, the main subtypes of CTCL include mycosis fungoides (MF), which accounts for approximately 60% of primary CTCL, and Sézary syndrome (SS), which accounts for approximately 5%. Notably, there are significant differences between these two subtypes in terms of their pathological evolution, immunophenotype, and clinical prognosis [4,5].

The initial disease progression of patients with MF is slow. In terms of clinical manifestations, these patients may experience numerous years of pruritic erythematous scaly patches or blocks, which can easily be confused with chronic eczema, psoriasis, and allergic contact dermatitis [6]. These similarities often lead to delays in the diagnosis of CTCL, and some patients with MF may progress to skin tumors, which can affect the lymph nodes and several internal organs [7]. Moreover, some patients with MF may undergo large cell transformation (MF-LCT) [8], and due to the lack of an efficient and targeted treatment, the prognosis of such patients is generally poor. Additionally, SS is a leukemia-like variant often accompanied by systemic immune abnormalities and has a poor prognosis, with a five-year survival rate of less than 25% for terminal patients [4].

Overall, there are many subtypes of CTCL, but each accounts for less than 2% of the overall patient population [2,3]. Although CTCL can be differentiated and diagnosed based on its clinical manifestations, disease course, pathology, and immunohistochemical analysis [1,9], the numerous disease subtypes and lack of specific markers complicate the clinical diagnosis and treatment of CTCL. Current CTCL treatments include topical medications such as corticosteroids, nitrogen mustard, and retinoids, as well as phototherapy and radiotherapy [4,10,11]. With the development of omics and sequencing technologies, more in-depth research has been conducted on the disease markers and pathogenic mechanisms of CTCL [12]. In particular, studies have shown that multiple genes are involved in T-cell activation, DNA repair, NF-κB activation, STAT3 activation, CCR4/CCR7 signaling, and JAK-STAT signaling [13,14,15,16], but the complex molecular regulatory mechanisms remain unclear, thus limiting targeted treatment options [5,17].

Notably, in recent years, chemokines have been proven to play a significant role in all stages of CTCL disease [18], as they mediate the innate and adaptive immune responses within the TME by recruiting immune cells to the tumor site [19]. Moreover, innovative biologic agents targeting chemokine receptors [4], such as CCR4 antagonists and CCR4 monoclonal antibodies [18,20], have shown promising safety and efficacy in clinical studies. However, patients with advanced MF and SS still respond poorly to most treatment regimens, which poses a severe challenge for the clinical management of CTCL [21]. As such, this article aims to systematically explore the dysregulation of the chemokine network in CTCL TME, clarify this network’s key role in shaping an immunosuppressive microenvironment, and review the progress of drug development targeting this network in order to highlight new research directions for understanding the pathogenesis of CTCL and developing precise treatment strategies.

## 2. Impact of Chemokine Interactions in CTCL

Chemokines are a class of small molecular proteins that mediate cell migration through G protein-coupled receptors (GPCRs) [22,23]. The primary function of chemokines is to regulate the migration and positioning of immune cells within tissues, which is crucial for maintaining immune system homeostasis [14,24]. Chemokines can be divided into four main families based on the arrangement of conserved cysteine residues: CC, CXC, CX3C, and XC (C; also known as the C family) [23,24]. Because current mechanistic and translational evidence in CTCL predominantly involves CC and CXC chemokines, we mainly focus on these two families. The interactions between chemokines and their receptors are highly complex, as most chemokines can bind to multiple receptors, and a single receptor can recognize multiple chemokines, thus forming a dynamic and finely regulated signaling network [25]. Numerous studies have shown that chemokines play a key role in the TME [26], as they not only participate in the recruitment of immune cells to the tumor site but also regulate the initiation of immune-inflammatory responses [27]. Notably, chemokines have a dual biological effect: they can mediate tumor metastasis and also activate anti-tumor immune responses and enhance the effects of immunotherapy [28]. Since this bidirectional regulatory effect relies on the complex network of the chemokine-receptor system [26], the in-depth analysis of the regulatory mechanisms underpinning the action of chemokines in the CTCL TME is necessary to establish a comprehensive understanding of the pathogenesis of CTCL and provide potential targets for treatment. To facilitate navigation, we provide an overview of the key chemokine–receptor axes grouped by their primary functional themes in CTCL (Table 1).

### 2.1. CC Chemokine Family

Key chemokine-receptor signaling axes within the CC chemokine family include CCL17/CCL22-CCR4, CCL18-CCR8, CCL19/CCL21-CCR7, CCL2-CCR2, CCL27/CCL28-CCR10, CCL20-CCR6, and CCL5-CCR5, which play significant roles in the immune regulation of CTCL. The specific functional mechanisms of these axes remain to be further elucidated [55,56]. In the following sections, current research on each of these signaling axes is systematically reviewed.

Among CC-family pathways, CCL17/CCL22–CCR4 represents a central axis in CTCL progression [19,29]. Specifically, this axes not only directly promotes the survival and proliferation of malignant T cells but also induces the functional polarization of tumor-associated macrophages (TAMs), thus establishing a self-enhancing immunosuppressive microenvironment that drives the continuous development of the disease [30]. C–C chemokine receptor 4 (CCR4) is highly expressed on the surface of malignant T cells [29] and can mediate the directional migration of these cells to skin lesions by binding to CCL17 and CCL22, derived from stromal cells and M2 macrophages [19,57,58]. Additionally, when CCL17 and CCL22 bind to the CCR4 receptor, they both further upregulate the expression of their own receptors and also activate the GATA-3 signaling pathway within malignant T cells [59], thereby enhancing the expression of interleukin-4 (IL-4) and interleukin-13 (IL-13). Subsequently, these cytokines promote the polarization of lymphoma-associated macrophages (LAMs) toward a specific functional phenotype. The polarized LAMs can also produce more CCL17 and CCL22, which forms a self-enhancing loop that continuously amplifies the CCL17/CCL22-CCR4 interaction [30]. Monoclonal antibodies targeting CCR4 can effectively eliminate CCR4^+^ malignant T cells in the peripheral blood and skin of patients; however, some patients may experience adverse reactions, such as drug-related rashes [60]. Serum CCL17 levels are positively correlated with skin tumor burden and are significantly higher in patients with tumor-stage MF compared to patients in the patch stage. Additionally, the levels of macrophage-derived chemokines in their microenvironment are also significantly correlated [53]. It is noteworthy that the high expression of CCL17 in CTCL is significantly different from psoriasis vulgaris, suggesting that it may serve as a potential biomarker for the differential diagnosis, disease monitoring, and treatment response assessment of CTCL [31,53].

In addition to CCR4 ligands, CCL18 and CCL26 are frequently elevated in CTCL serum, and lesions and have been associated with disease progression and adverse prognosis [50,51,61]. Indeed, the CCL18 serum levels are approximately threefold higher in MF patients compared to healthy controls. This chemokine has been confirmed to have immunomodulatory effects on CTCL. Indeed, CCL18 can promote the chemotactic migration of CTCL cells, and it also inhibits the in vitro growth of CTCL cell lines to a certain extent [51]. By binding to its CCR8 receptor, CCL18 mediates the transmission of inhibitory signals from CCL13^+^ monocytes/macrophages and LAMP3^+^ cDCs to malignant T cells, thereby promoting the establishment of an immunosuppressive microenvironment [33]. Furthermore, the expression of CCL18 was also found to be upregulated in lesion tissues of atopic dermatitis and bullous pemphigoid, suggesting that these diseases may share similar immune regulatory pathways or upstream regulatory mechanisms [50]. CCL26 is thought to reflect a Th2-skewed cytokine milieu in CTCL lesions, potentially reinforcing Th2-associated inflammation and thereby correlating with disease progression and poor prognosis [56,61,62].

CCL19/CCL21–CCR7 is a key chemotactic axis involved in lymphocyte homing and may contribute to malignant T-cell dissemination in CTCL. CXCL13 has been reported to synergize with CCL19/CCL21 through CCR7, markedly enhancing the chemotaxis and migratory capacity of SS cells [32,63]. The activation of CCR7 has also been confirmed in patients with MF. Specifically, CCR7 can promote the migration of MF cells by activating the mTOR signaling pathway [64]. Single-cell sequencing further identified CCR7 as the main surface marker of a malignant T cell subpopulation, indicating that CCR7 is a key chemotactic factor receptor that mediates lymphocyte homing and migration and, thus, —may play an important role in the disease regulation of CTCL [34]. Single-cell sequencing data allows for the expression regulation network and functional mechanisms underpinning the action of CCR7 to be more accurately analyzed, thus providing high-resolution evidence for subsequent research on this topic [36,49].

The CCL2–CCR2 axis contributes to TME construction primarily by promoting recruitment of monocytes/macrophages into lesions [37,38]. RNA-Seq analysis revealed that, in the CTCL TME, the chemokine CCL2 and its receptor CCR2 were highly expressed in M2-type TAMs. This axes promotes the recruitment of peripheral monocytes and macrophages to the tumor tissue, thus forming an immunosuppressive microenvironment dominated by TAMs [38]. Previous studies have found that using CCR2 inhibitors can significantly reverse the enrichment of macrophages in the tumor tissue, effectively reduce the tumor burden of CTCL, and thus, demonstrate good anti-tumor activity. However, the detailed molecular and cellular mechanisms by which the CCL2-CCR2 axes mediates these effects have not been fully elucidated and require further in-depth research. Indeed, clarifying these mechanisms is expected to provide new targets and strategies for CTCL immunotherapy [37].

Another two chemokines, CCL27 and CCL28, together with their shared receptor CCR10, are significantly overexpressed in MF and SS, which facilitates the homing of malignant T cells to the skin [39,40,65,66]. Notably, serum CCL27 levels show significant changes from before to after treatment in CTCL patients, suggesting that CCL27 could serve as a potential biomarker for the dynamic monitoring of disease progression [54]. Meanwhile, several studies have confirmed that its receptor, CCR10, is specifically overexpressed in CTCL. This differential chemokine and receptor expression pattern compared to other inflammatory diseases also provides additional support for the differential diagnosis of CTCL [65].

Another signaling axes from the CC family, CCL20-CCR6, plays a key role in the metastatic process of CTCL [52]. In particular, the continuous activation of the STAT3 transcription factor within CTCL cells directly drives the transcription and expression of CCL20 via autocrine secretion. This CCL20 then binds to CCR6 on the cell surface, forming a self-reinforcing signaling loop that promotes tumor cell invasion and migration [52]. In a late-stage CTCL mouse model, intervention with miR-150 targeting CCR6 effectively downregulated the expression of CCL20, disrupted this autocrine loop, significantly inhibited CTCL metastasis to distant organs, and thus, extended the survival of the model mice [45,67].

Finally, current research on the role of the CCR5 pathway in CTCL remains relatively limited. CCR5 with its ligand CCL5 has been implicated as a context-dependent axis in MF, as transcriptomic/proteomic analyses show that Vorinostat can differentially modulate CCL5/CCR5 expression across MF cell lines, highlighting disease heterogeneity and a potential link to treatment-responsive immune signaling programs [68]. The existing evidence indicates that CCR5 is highly expressed in some CTCL skin biopsy samples [39], and RNA-seq data derived from these biopsies further revealed the expression of CCR5 in M2-type TAMs within the TME [38]. These preliminary findings suggest that CCR5 may be involved in the pathological process of CTCL, although the precise functional mechanisms of this association remain to be elucidated. The in-depth investigation of CCR5 is still in its early stages, and its potential pathogenic roles and value as a therapeutic target require further exploration. The major CC chemokine–receptor axes involved in CTCL are listed in Table 2, illustrating the diverse roles of these pathways in regulating malignant T-cell migration, immune modulation, and disease progression.

### 2.2. CXC Chemokine Family

The CXC chemokine family and their receptors have been extensively studied in solid tumors and hematological malignancies, particularly regarding their roles in immune regulation and tumor invasion mechanisms [46]. In contrast, studies addressing the CXC chemokine family in CTCL remain relatively limited. Current evidence indicates that CXC chemokine axes participate in malignant T-cell migration, skin homing, TME modulation, and disease progression [41,47]. The most widely investigated signaling axes include CXCL9/10/11–CXCR3, CXCL13–CXCR5, and CXCL12–CXCR4, discussed as below.

CXCR3 has attracted particular attention in the CXC chemokine family. Multiple studies have focused on the immunoregulatory functions of its ligands CXCL9, CXCL10, and CXCL11 in CTCL [46]. These CXC chemokines, typically induced by IFN-γ, recruit activated T cells and NK cells to sites of infection or tumor development, thereby mediating anti-tumor immune responses [70]. CXCR3 is highly expressed in the epidermis of CTCL patients but tends to be lost during large-cell transformation in MF. In other cutaneous lymphomas, CXCR3 expression has been detected in four cases of lymphomatoid papulosis, four cases of CD8^+^ CTCL, and three of six cases of systemic CTCL, whereas it was absent in 10 cases of cutaneous anaplastic large-cell lymphoma [48]. These findings suggest CXCR3 contributes to epidermotropic localization of malignant T cells and may influence tumor phenotype [48].

Further research on CXCR3-dependent T-cell migration revealed that CXCR3 not only participates in TME remodeling but also correlates with immune escape mechanisms. CXCR3 may play a dual role in CTCL progression, mediating effector T-cell trafficking and promoting an immunosuppressive milieu through immune-cell interactions [41]. In treated CTCL skin lesions, CXCL9 and CXCL11 are markedly upregulated, primarily secreted by infiltrating macrophages, which promote the accumulation of CXCR3^+^ effector T cells within the skin and establish a distinct TME [18]. This pathway has been associated with both long-term remission and treatment-related adverse events [18,42]. Although CXCL13–CXCR5 signaling has also been implicated in CTCL, its precise mechanistic role remains to be determined [42]. Notably, dynamic alterations in CXCL9, CXCL10, and CXCL11 expression within the TME may reflect the plasticity between Th1 and Th2 cells, contributing to disease evolution [43]. Collectively, the CXCR3 axes appears to exert context-dependent effects, regulating immune-cell migration, differentiation, and activation during CTCL pathogenesis.

CXCR4–CXCL12 axes play a central role in the directional chemotaxes of lymphocytes [44]. In SS, high CXCR4 expression facilitates the precise migration of malignant T cells to the skin, enabling skin-homing behavior. Functional studies demonstrate that pharmacologic inhibition of CXCR4 for example, Bortezomib can significantly reduce CTCL cell migration and decrease tumor-cell survival [71]. During MF progression, CXCR4 and CXCL12 expressions increase concomitantly as the disease advances from the plaque to the tumor stage, reinforcing the crucial involvement of this signaling axes in CTCL development and progression [72]. Moreover, the CXCR4–CXCL12 pathway contributes to cell proliferation and angiogenesis, suggesting it may further enhance CTCL progression by augmenting tumor-cell growth and TME vascularization [73].

In conclusion, chemokine receptor signaling networks constitute the central regulatory hub of the dynamic TME in CTCL. Chemokines orchestrate malignant T-cell colonization and migration in the skin and shape the immune landscape by directing immune-cell recruitment, differentiation, and suppression. Furthermore, CTCL cells themselves secrete immunomodulatory chemokines such as CCL5 and CCL22, which attract regulatory T cells and M2-polarized macrophages, fostering a highly immunosuppressive microenvironment that promotes immune evasion and sustained tumor progression. Future studies exploring these signaling networks are essential for developing targeted therapies aimed at disrupting chemokine-mediated immunosuppression in CTCL. The major CXC chemokine receptor signaling axes implicated in CTCL pathogenesis are summarized in Table 3.

### 2.3. CX3C and XC Chemokine Families

In addition to CC and CXC chemokines, the chemokine system also includes the CX3C and XC (C) families. The CX3C family contains a single member, CX3CL1 (fractalkine), which binds CX3CR1 and can exist in both membrane-bound and soluble forms, thereby functioning in leukocyte adhesion and chemotaxis [74,75]. The XC family includes XCL1 and XCL2, which signal through XCR1 and are primarily linked to dendritic cell–mediated cross-presentation and cytotoxic T-cell priming [76].

At present, direct evidence connecting CX3C and XC chemokines to CTCL pathogenesis and TME remodeling remains limited compared with the well-characterized CC and CXC axes. Therefore, these two families are not discussed in depth in this review; however, we highlight them as potentially relevant pathways that may be revealed by future single-cell and spatial-omics studies in CTCL.

In conclusion, chemokine–receptor axes serve as key regulators of malignant T-cell trafficking and immune-cell positioning in CTCL. Section 2 summarizes the major dysregulated axes and their primary functional implications at the axis level. The integrated, network-level consequences of these axes—particularly their coordinated roles in shaping an immunosuppressive CTCL tumor microenvironment—are discussed in Section 3.

## 3. Construction of the CTCL TME Chemokines

**Figure 1:**
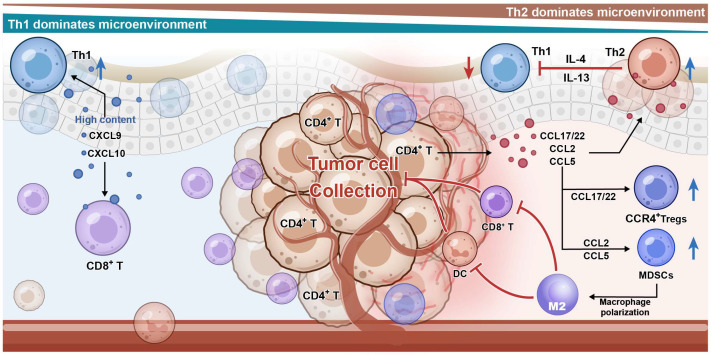
Chemokine–receptor networks drive stage-dependent remodeling of the CTC-TME. In the tumor microenvironment of cutaneous T-cell lymphoma (CTCL), malignant T cells act as a central hub, actively shaping a protective, immunosuppressive niche for themselves. Malignant T cells secrete key chemokines, including CCL22, CCL2, and CCL5. These chemokines form concentration gradients that recruit various immunosuppressive cells: regulatory T cells (Tregs) (via the CCL22-CCR4 axis), myeloid-derived suppressor cells (MDSCs) (via the CCL2-CCR2 axis), and M2-polarized macrophages (via the CCL5-CCR5 axis). These cells form a physical and functional barrier around the tumor cells. This barrier facilitates immune evasion by excluding and inhibiting effector immune cells (e.g., CD8^+^ T cells, NK cells), leading to their functional impairment and creating an immunosuppressive milieu within the microenvironment.

The TME is considered a key regulatory factor in the development and progression of CTCL [77]. The CTCL TME comprises not only malignant T cells but also various immune and non-immune cellular components such as dendritic cells, regulatory T cells (Tregs), myeloid-derived suppressor cells (MDSCs), TAMs, and cancer-associated fibroblasts (CAFs) [78,79,80]. Additionally, various signaling molecules play important roles in the TME, including chemokines, cytokines, and metabolites [81]. The chemokine network represents a multi-layered immunosuppressive niche in the CTCL TME with spatiotemporally specific intercellular communication [82]. In this network, different types of immunosuppressive cells are recruited and undergo functional transformation, which provides support for the survival and immune evasion of malignant T cells [79]. This dynamic regulatory chemokine network not only promotes the proliferation and migration of tumor cells but also profoundly affects their interaction with the host immune system [62]. The chemokine-mediated immunosuppressive loop in CTCL is summarized in Figure 1, highlighting how malignant T cells act as both the source and the beneficiary of a suppressive microenvironment. Through the coordinated secretion of CCL22, CCL2, and CCL5, they attract Tregs, MDSCs, and M2 macrophages, forming a feedback circuit that maintains immune evasion and sustains disease progression.

### 3.1. Immune Polarization from Th1 to Th2

The TME of CTCL exhibits significant immune remodeling characteristics depending on the disease stage [82]. In particular, in the early-stage lesions are often characterized by a more Th1-like inflammatory context, whereas progression to advanced disease is associated with a shift toward Th2-skewed chemokine and cytokine programs, including increased Th2-related chemokines (CCL17/CCL18/CCL22/CCL26) and attenuation of Th1-associated chemokines (CXCL9/10/11) [78,83]. In the Th2-dominated microenvironment, cytokines released by Th2 cells, such as IL-4 and IL-13, can significantly inhibit Th1 cell differentiation and immune resistance. This transition can weaken cytotoxic immune surveillance and promote an immunosuppressive milieu that supports malignant T-cell persistence and expansion [33].

Single-cell RNA sequencing studies have identified lesion-enriched dendritic cell and myeloid populations expressing chemokines such as CCL17, and these populations may facilitate CCR4-dependent interactions between malignant T cells and Tregs, contributing to immunosuppression [33]. This imbalance in the chemokine network leads to multiple forms of immunosuppression [33]. CCL18 is expressed by monocytes and dendritic cells and is significantly elevated in the skin and serum of CTCL patients. CCL18 can participate in the recruitment of benign infiltrating Th2 cells [50]. The increase in the number of benign Th2 cells in the microenvironment can enhance the inflammatory response and provide signals for tumor growth [50]. LIGHT-HVEM, as an important Th1 chemokine pathway, promotes the production of large amounts of CXCL9, CXCL10 and CXCL11 in the early skin of CTCL. However, in the late skin of CTCL, the low expression of HVEM reduces the expression of TH1 chemotactic factors, leading to the transformation of TME to Th2 [84].

Recent studies suggest that a Th2-skewed chemokine signature in the TME (including elevated CCR4 ligands such as CCL17/CCL22) may correlate with reduced benefit from immune checkpoint blockade in CTCL, consistent with the concept that chemokine-driven immune exclusion and regulatory programs can dampen effective anti-tumor immunity. These findings support exploring rational combinations that couple Treg-myeloid reprogramming with checkpoint inhibitors.

### 3.2. Recruitment of Immune Cells by Malignant T Cells

During CTCL progression, a large number of immune cells are recruited to the tumor surface, a process primarily led by malignant T cells [85]. Research shows that malignant T cells can recruit CCR1-expressing myeloid immune cells to the tumor site by secreting CCL5 and CCL23 [86]. The increase in the number of local myeloid immune cells enhances the secretion of pro-inflammatory cytokines such as IL-1β, IL-6, and IL-15, thereby promoting tumor growth [86]. This indicates that in CTCL, malignant T cells are not merely responders to regulatory factors like cytokines but are active constructors shaping the TME. Furthermore, myeloid immune cells can also recruit CCR10-expressing T cells to the skin surface by secreting CCL2 [86]. In the pro-inflammatory TME, tumor cells can proliferate rapidly, and the local inflammation caused by cytotoxic reactions also causes significant pain to patients.

A Treg-enriched immunosuppressive milieu is a recurrent feature of CTCL lesions and can be reinforced by local chemokine gradients. Within lesional skin, CCR4 ligands (CCL17/CCL22) can promote the recruitment and retention of CCR4^+^ Tregs. In advanced disease, CXCL12 may further support the localization of CXCR4^+^ regulatory and malignant populations [29]. Functionally, heightened Treg activity can suppress dendritic-cell maturation and dampen effector CD8^+^ T-cell function through mechanisms involving CTLA-4 engagement and immunosuppressive cytokines such as IL-10 and TGF-β.

Single-cell transcriptomic studies indicate CTCL lesions harbor multiple Treg states [87]. These include effector-like FOXP3^hi populations enriched for suppressive (and in some contexts cytotoxic) programs, as well as naïve-like FOXP3^low populations with differentiation-associated features [88,89]. In peripheral blood, expansion of Treg-like populations has been reported in SS and may be associated with reduced responsiveness to immune checkpoint blockade [90]. Together, these observations underscore the value of integrating lesional and blood immune profiling when evaluating or optimizing immunotherapy strategies.

By secreting CCL2, CCL5, and CCL18, malignant T cells can recruit MDSCs and M2-type macrophages to the CTCL TME [38]. Among these recruited cells, the CCR2^+^ monocytes recruited by CCL2 differentiate into MDSCs with immunosuppressive function or M2-type TAMs in the TME. Similarly, CCL5 recruits CD14^+^HLA-DR^−^/low MDSCs and CX3CR1^+^ immunosuppressive monocytes that express CCR5 on their surface [52]. These monocyte cells deplete essential amino acids, such as L-arginine, in the local microenvironment and disrupt T-cell receptor signaling by producing mediators such as arginase-1 (Arg-1) and reactive oxygen species (ROS), ultimately leading to CD8^+^ T cell dysfunction [91]. As the TME is remodeled by malignant T cells, it gradually loses its immune surveillance function, which, in turn, provides favorable conditions for tumor growth and metastasis [15]. As the tumor progresses, CCL18 released by M2 macrophages can further recruit inhibitory cells, thus forming a self-amplifying immunosuppressive cycle within the TME. Together, MDSCs and M2-type TAMs are the key components of immunosuppression in the CTCL TME, as they effectively suppress anti-tumor immune responses and provide protection for malignant clone expansion [92].

The development of high-throughput omics technologies, particularly next-generation sequencing (NGS) technologies, has allowed the molecular mechanisms and the TME of CTCL to be understood in more detail [93]. For instance, previous studies have found that MF and SS originate from different T-cell subsets, namely effector memory T cells (Tem) and central memory T cells (Tcm), respectively. This difference not only reflects their varying migration capacities and antigen recognition characteristics but also highlights their significant differences in treatment sensitivity and recurrence risk. Through single-cell sequencing, studies have identified a large number of T-cell subsets in CTCL patients expressing markers such as CXCR3^+^, GNLY^+^, CREM^+^, and MKI67^+^ [36]. These T cells are often associated with high proliferation indices, stem cell-like phenotypes, and chromosomal copy number variations (CNV), suggesting they are tumor stem cell-like populations that play a key role in the continuous evolution of the disease.

In summary, CTCL is not only a clonal T-cell malignant tumor driven by genetic mutations but also a chronic immune tumor strongly regulated by the immune microenvironment. Changes in the chemokine network are an important factor driving TME remodeling and also create favorable conditions for tumor survival, migration, and immune escape. Therefore, systematically analyzing the expression patterns and functional roles of CTCL-related chemokines at different disease stages is necessary to reveal their immune regulation mechanisms and provide new targets for clinical intervention.

## 4. Therapeutic Strategies Targeting Chemokines

Based on the crucial role of chemokines in the construction of the CTCL TME and tumor promotion, targeting the chemokine network and its complex regulatory mechanisms represents a highly attractive therapeutic strategy. In recent years, interventions targeting this network have been developed that aim to block the migration and homing of malignant T cells and disrupt the immunosuppressive microenvironment, thereby supporting effective anti-tumor immunity, alleviating clinical symptoms, and improving patient survival.

### 4.1. CCR4 Inhibitor—Mogamulizumab

Mogamulizumab is a humanized monoclonal antibody that works against CC chemokine receptor 4 (CCR4) through a dual mechanism of action [94]. Mogamulizumab was approved by the EMA in 2018 for CTCL patients who have received at least one prior systemic therapy [95], and it is positioned in most subsequent guidelines as a systemic option for advanced or widespread disease (generally stage IIB–IV), often as a preferred/Category A regimen in the NCCN framework due to its relatively favorable toxicity profile. Across updated national guidelines, Mogamulizumab is commonly recommended as a second-line systemic therapy for MF/SS after failure of classic agents, with caution advised around allogeneic stem-cell transplantation because prior MOG exposure may increase the risk of post-transplant GVHD [96,97].

First, Mogamulizumab directly clears CCR4-positive malignant T cells and Tregs through antibody-dependent cellular cytotoxicity (ADCC), and second, it blocks the binding of CCL17 and CCL22 to CCR4, thus inhibiting the migration of malignant T cells to the skin and the recruitment of Tregs [38]. The pivotal phase III MAVORIC trial confirmed that, compared with Vorinostat, Mogamulizumab significantly improved progression-free survival and overall response rate in patients with relapsed or refractory MF and SS; additionally, Mogamulizumab showed especially pronounced efficacy in patients with blood involvement [20,98]. This finding has made Mogamulizumab the first CTCL treatment drug targeting a chemokine receptor that is approved in multiple countries.

However, common side effects of Mogamulizumab include infusion reactions and drug-associated rash, the latter of which may be related to the redistribution and activation of effector T cells in the skin [99,100]. Real-world studies and multicenter retrospective cohorts generally confirm Mogamulizumab clinical activity in heavily pretreated CTCL, often showing higher response rates in SS and in the blood and skin compartments (with reported overall response rates frequently in the ~40–60% range in larger series). Across these reports, treatment is usually tolerable, with rash and infusion-related reactions as the most common adverse events, and mogamulizumab-associated rash has been repeatedly linked to better clinical responses, although durability can vary and resistance may emerge in a subset of patients [101,102,103]. A more severe challenge is the emergence of drug resistance through mechanisms involving target downregulation, the compensatory activation of other signaling pathways, and weakened ADCC effects due to NK cell functional exhaustion [104].

Besides Mogamulizumab, other CCR4 small molecule antagonists (AZD-2098) are in development [105]. Compared with monoclonal antibodies, small molecule drugs may have better tissue penetration and be less likely to cause ADCC-related side effects, but most are still in the preclinical or early clinical stages. Strategies that focus on neutralizing antibodies and targeting CCR4 ligands (CCL17 and CCL22) to “cleanse” the microenvironment have been explored for other cancer types, but their potential for application in CTCL still requires validation.

In terms of combination therapy, using CCR4 inhibitors and immune checkpoint inhibitors (ICIs) together has shown synergistic potential [106]. Specifically, Mogamulizumab depletes Tregs to relieve immunosuppression, thus creating favorable conditions for PD-1/PD-L1 inhibitors to reactivate effector T-cell function and forming a de-inhibition and reactivation treatment model. In this context, several early-phase clinical trials are currently evaluating the safety and efficacy of the combination of Mogamulizumab and Pembrolizumab (an anti-PD-1 antibody) [107,108].

### 4.2. CXCR4—Canonical Antagonism and Indirect Modulation

Plerixafor is a canonical CXCR4 antagonist approved for hematopoietic stem-cell mobilization in patients with non-Hodgkin’s lymphoma and multiple myeloma [109,110]. By disrupting CXCL12–CXCR4 binding, CXCR4 antagonism may, in principle, interfere with malignant T-cell trafficking and retention programs relevant to CTCL. However, direct clinical evidence supporting CXCR4 antagonists in CTCL remains limited, and translation will require a careful evaluation of efficacy, patient selection, and potential on-target effects given the broad physiological roles of CXCR4. Importantly, some agents may reduce CXCR4 expression indirectly; for example, the proteasome inhibitor Bortezomib has been reported to downregulate CXCR4 and immunosuppressive cytokines in CTCL cell systems, an effect distinct from direct receptor antagonism [71].

### 4.3. Other Emerging and Preclinical Chemokine-Directed Strategies

Targeting CCR2 and CCR5 may help attenuate myeloid recruitment and TAM/MDSC-associated programs in CTCL. The CCL2–CCR2 axis is implicated in macrophage recruitment in CTCL lesions, and preclinical studies suggest that CCR2 antagonism can reduce TAM enrichment and may synergize with immune checkpoint blockade in CTCL models [111]. The CCL5–CCR5 axes has also been linked to recruitment of immunosuppressive myeloid populations, but CTCL-specific clinical evidence remains limited. Because CCR2/CCR5 are broadly expressed across monocyte/macrophage lineages, potential toxicities should be considered when translating broad myeloid-targeting strategies [112].

CCR8 has been implicated in regulatory programs that may contribute to CTCL progression. Single-cell studies implicate CCR8-associated myeloid–lymphoid communication programs in CTCL immunosuppression [113]. While CCR8 is being explored as an immuno-oncology target in other malignancies (often with the goal of modulating tissue Tregs) [114], CTCL presents unique considerations because CCR8-related programs may intersect with malignant T-cell biology; therefore, CTCL-specific validation is needed.

CCR6 represents a tumor-intrinsic invasion axis in advanced CTCL. Invasion and metastasis can be driven by an IL-22–STAT3–CCL20–CCR6 cascade, in which STAT3-dependent CCL20 reinforces CCR6-associated migratory programs [115]. Restoring tumor-suppressive miRNAs (e.g., miR-26 and previously miR-150) or suppressing IL22/IL22RA disrupts this pathway, inhibits CTCL cell migration, and promotes apoptosis in vitro. Consistently, in vivo IL22 knockdown abrogates tumor invasion and metastasis and prolongs survival. Collectively, these findings highlight the IL-22–STAT3–CCL20–CCR6 axis as a compelling preclinical vulnerability, although pharmacological translation remains at an early stage [115]. All the major chemokine receptor-targeted therapeutic strategies for CTCL, including CCR4, CXCR4, CCR5, and CCR6 modulators, are summarized in Table 4.

## 5. Conclusions

CTCL is characterized by significant heterogeneity in its clinical manifestations and biological processes, and the complexity of its molecular pathogenesis and the regulatory networks within the TME present a formidable challenge for the systematic understanding of this disease [112]. The cytokine signaling network, which encompasses both chemokines and inflammatory factors, contributes a further layer of complexity due to its intricate regulatory mechanisms and inherent functional redundancy. While emerging technological approaches have enabled the higher-resolution investigation of chemokine networks in the TME, this functional redundancy remains a major translational obstacle. For instance, the inhibition of a single receptor may trigger the compensatory upregulation of alternative receptors or increased ligand secretion [24], thereby sustaining an immunosuppressive state and leading to therapeutic resistance. Furthermore, much of the current evidence relies on bulk sequencing or tissue homogenization techniques. Problematically, these techniques obscure the precise cellular sources and subset-specific distribution of chemokines, which are a critical prerequisite for unraveling their regulatory mechanisms. As such, establishing dynamic models of immune phenotype transitions and effectively integrating disease-stage biomarkers with clinical management strategies are ongoing challenges in CTCL research.

Importantly, the development and implementation of NGS, particularly single-cell technologies, has begun to transform this research landscape. Indeed, researchers can now obtain high-resolution, cell-specific expression data, enabling the detailed mapping of the cellular atlas in the CTCL TME, the identification of comprehensive chemokine expression profiles, and the analysis of their correlations with tumor behavior. With these capabilities, there is an unprecedented opportunity to elucidate the mechanisms by which chemokines promote disease progression through regulating tumor cell migration, proliferation, and immune evasion. Additionally, chemokines are not only pivotal regulators of the CTCL TME but also hold substantial promise as disease biomarkers and therapeutic targets. Research targeting receptors such as CCR4 and CXCR4 has advanced considerably; for example, the anti-CCR4 monoclonal antibody Mogamulizumab has demonstrated significant clinical efficacy in a subset of patients and represents a new paradigm for precision therapy [20].

In terms of future recommendations, overcoming the current limitations of this evidence base will require a concerted multi-modal approach. Future research must leverage single-cell sequencing integrated with spatial transcriptomics and proteomics to accurately delineate intercellular communication networks. This understanding will clarify the context-specific modes of action within the chemokine network and provide a robust technical foundation for developing precise clinical biomarkers and novel therapeutic targets. The integration of serum chemokine levels, peripheral blood immune phenotypes, and tumor tissue immune profiling would also be useful for the construction of predictive models aiming to achieve rapid patient stratification and individual target prioritization as part of a personalized medicine approach.

Despite the recent advances in the understanding of CTCL, significant obstacles remain. Maintaining therapeutic specificity within the complex, redundant TME and addressing significant inter-patient heterogeneity demand more sophisticated analytical frameworks. These challenges highlight the need for enhanced data analysis and interpretation capabilities when using emerging technologies. Consequently, future efforts must combine multi-omics data integration with rigorous functional validation experiments to comprehensively reveal the mechanisms of action of key cytokines and explore their practical utility in precision treatment strategies. Ultimately, interdisciplinary collaboration will be paramount in translating these foundational insights into clinical practice and paving the way to effective precision-targeted therapies for CTCL.

## Figures and Tables

**Table 1:** Functional grouping of key chemokine–receptor axes in CTCL (overview).

Functional Theme	Key Chemokine Receptor Axes	Axis-Level Relevance in CTCL
Skin homing and tissue trafficking	CCL17/CCL22–CCR4	Supports preferential skin tropism and lesion localization of malignant T cells; frequently highlighted across CTCL stages [19,29,30,31].
	CCL19/CCL21–CCR7	Associated with malignant T-cell chemotaxis and migration capacity; may mark more aggressive malignant subsets [32,33,34,35].
	CCL27/CCL28–CCR10	Facilitates skin homing and may support clinical monitoring via dynamic serum changes [36,37,38,39,40].
	CXCL12–CXCR4	Promotes directional chemotaxis/skin trafficking and is linked to disease progression [41,42,43,44].
	CXCL9/10/11–CXCR3	IFN-γ–inducible axis linked to epidermotropic localization and context-dependent immune cell trafficking [18,45,46,47,48].
Immunosuppressive niche formation	CCL2–CCR2	Enriched in CTCL lesions/TME and associated with monocyte/macrophage recruitment in CTCL-related settings [36,49].
	CCL5–CCR5	Evidence suggests CCR5 expression in some CTCL skin samples and in M2-like TAMs; functional mechanisms remain under investigation [36,37].
	CCL18–CCR8	Links myeloid/DC compartments to malignant T cells via CCR8-mediated signaling; implicated in immunoregulatory programs [35,50,51].
	CCL20–CCR6	STAT3-driven axis associated with invasion/metastasis features in advanced CTCL models [52].
Biomarker candidates	CCL17	Serum level correlates with tumor burden and may help differential diagnosis/monitoring [53].
	CCL18	Elevated in serum/lesions and associated with severity/prognosis; immunomodulatory activity reported [50].
	CCL27	Serum levels may change with treatment and support dynamic monitoring [54].
	CXCL9/10/11	Stage and treatment-context signals reflecting immune polarization and trafficking dynamics [18].

**Table 2:** Major CC chemokine receptor axes involved in CTCL.

Chemokine Receptor Axis	Brief Function Summary
CCL17/CCL22—CCR4	Drives malignant T-cell skin homing and supports an immunosuppressive loop involving macrophage polarization and Treg recruitment [19,29,30,53].
CCL18—CCR8	Associated with disease progression and immunosuppressive signaling in the CTCL microenvironment [33,50,51,61].
CCL26-CCR3	Elevated in advanced disease and associated with poor prognosis [56,61,62,69].
CCL19/CCL21—CCR7	Enhances malignant T-cell chemotaxis/migration; CCR7 marks aggressive malignant subsets [32,33,62,63].
CCL2—CCR2	Recruits monocytes/macrophages and promotes TAM-dominated immunosuppressive TME [37,38].
CCL27/CCL28—CCR10	Mediates skin homing of malignant T cells; CCL27 may help monitor treatment response [39,40,54,65,66].
CCL20—CCR6	STAT3-driven autocrine loop promoting invasion and metastasis [45,52,67].
CCL5—CCR5	Potentially contributes to recruitment of suppressive myeloid populations; mechanisms remain under investigation [68]

**Table 3:** Major CXC chemokine receptor axes involved in CTCL.

Chemokine Receptor Axis	Brief Function Summary
CXCL9/10/11—CXCR3	Recruits activated T and NK cells; contributes to anti-tumor immunity and shows context-dependent effects during CTCL evolution [18,42,43].
CXCL13—CXCR5	Implicated in malignant T-cell migration (especially in SS), though precise mechanisms remain unclear [42].
CXCL12—CXCR4	Drives malignant T-cell chemotaxis/skin trafficking and supports proliferation and angiogenesis; associated with disease progression [44,71,72,73].

**Table 4:** Summary of therapeutic strategies targeting the chemokine network in CTCL.

Target Axes	Representative Approach	Key Mechanism	Evidence Level in CTCL
CCR4	Mogamulizumab	ADCC-mediated depletion of CCR4^+^ malignant cells/Tregs; blocks CCL17/CCL22 binding	Phase III efficacy; regulatory approval [20,116]
CXCR4	Plerixafor	Canonical CXCR4 antagonism; blocks CXCL12 binding	Rationale based on expression/biology [109,110]
CXCR4	Bortezomib	Proteasome inhibition; indirect downregulation of CXCR4 and cytokines	In vitro/biologic evidence [71]
CCR2	CCR2 antagonists	Reduces monocyte/TAM recruitment; may synergize with ICI	Preclinical [111]
CCR5	Maraviroc	Blocks CCR5-dependent recruitment/trafficking	Limited clinical observation [112]
CCR6	miR-150 restoration/miR-26	Disrupts CCL20–CCR6 autocrine invasion circuit	Preclinical [110]

## Data Availability

No new data were created or analyzed in this study. The original contributions presented in this study are included in the article. Further inquiries can be directed to the corresponding author(s).
